# New Appliances and Things Medical

**Published:** 1894-04-14

**Authors:** 


### NEW APPLIANCES AND THINGS MEDICAL.

[All preparations, appliances, novelties, &c., of which a notice is desired, should be sent for the Editor, to care of The Manager, 428, Strand, London, W.C.]

### PURE WHISKY.

(David Lennox and Co., 41, Pall Mall, S.W.)

The above well-known firm of distillers have forwarded us specimens of several of their brands of Whisky, namely 1, Pure Malt Whisky; 2, Old Highland Whisky; 3, Special Old Highland Whisky; and 4, Very Choice Old Highland Whisky. All these samples are excellent of their kind, uniform in quality, and prepared from pure malt. There is unfortunately at the present day a prejudice both in hospital and private practice to prescribe in those cases where alcohol is indicated that form of spirit which goes under the name of brandy. In a very large number of cases this means a doctored sample of potato spirit containing an undue proportion of amyl alcohol and other alcohols other than ethyl, all of which have an injurious effect on the system. It is a far safer practice to order a whisky from a well-known and responsible firm, than a brandy of doubtful origin and unreliable therapeutic effect.

### PILSENER LAGER BEER.

(O. Bruster and Co., 143a, Holborn, E.C.)

Samples of this beer were forwarded us some time ago, and although it has been kept the whole time at an equitable temperature of about 60 deg. Fah. it has shown no signs of falling off in condition; in fact it is as good a beer to-day as it was many weeks ago. This we consider an excellent result for a beer of so low a percentage of alcohol, and which so far as we can detect, is free from added preservatives. The beer appears to us to be an excellent sample of the class to which it belongs, namely a light German beer. It has an agreeable taste of the hops. Its use is particularly indicated for gouty and rheumatic subjects who enjoy their glass of beer but are unable to take the stronger English varieties.

### COCOA ESSENCE AND BON-BONS ASSORTIS.

(John Rothwell, Wigan.)

Messrs. Rothwell, of Wigan, have submitted a sample of their cocoa essence for our inspection. It is a cocoa of very agreeable flavour, and owing to its solubility is an exceedingly easy cocoa to mix. Their directions on the box for preparing for use are, according to our opinion, most inadequate. To begin with, no cocoa made entirely with water can be an agreeable beverage, and if made with the addition of milk is best made according to the following receipt: To make one pint of cocoa take three-quarters of a pint of milk, and boil; mix the dessert spoonful of cocoa with cold water into a smooth paste, and dilute up to a quarter of a pint. As the milk begins to boil up throw in the cocoa, and allow to boil again. The addition of three drops of essence of vanilla converts this cocoa into what is a really most excellent chocolate. For invalids cocoa is often a much more suitable beverage than tea or coffee, since in addition to its refreshing properties it is a food of considerable nutritive merit. There is a popular belief that it is rich and indigestible, a reputation which it scarcely deserves, and we have often given it to those recovering from gastric ulcer with unqualified success. There are of course people with idiocyncracies of digestion, and this applies to the tolerance of cocoa as well as to other food stuffs and drugs, and hence in a few individual cases shows that cocoa must be withheld. Our analysis shows that this essence of cocoa contains a very small percentage of fat, namely, 12 per cent., which figure, when compared to most other samples of cocoa, shows that Messrs. Rothwell must take considerable pains in the manufacture of their speciality. We find no admixture with foreign starches and sugars, and although we have not made a direct analysis of the amount of theobromine, the large percentage of ash and its richness in phosphoric acid are indicative of an unusually high percentage of this alkaloid. We can confidently recommend it as a pure and wholesome cocoa for general use or for invalids. Analysis: Moisture, 8·42 per cent.; fat, 12·18 per cent.; ash, 5·92 per cent.; ash containing phosphoric hydriode, 1·343. Excellent as is the cocoa supplied by this firm, we cannot but feel that they do themselves an injustice; firstly, by their directions for use, and, secondly, by their issue of "Bon-bons Assortis." These are sweets which for the most part are supposed to be chocolate. They have, however, so strong a flavour of cocoa and so little of vanilla that they are little likely to appeal to the general taste. We believe them, however, to be perfectly pure, which is more than can be said for a large number of sweetmeats in the market.

